# Survival of retinal ganglion cells in transgenic mice with deficiencies in sialyltransferases or neural cell adhesion molecule (NCAM) or after the administration of neuraminidase

**DOI:** 10.1186/2193-1801-4-S1-P24

**Published:** 2015-06-12

**Authors:** Natalia Lobanovskaya, Tamara Zharkovsky, Külli Jaako, Monika Jürgenson, Anu Aonurm-Helm, Alexander Zharkovsky

**Affiliations:** University of Tartu, Estonia

**Keywords:** PSA-NCAM, Sialyltransferase, Retinal ganglion cell

Neural cell adhesion molecule (NCAM) plays important roles in the regulation of the brain plasticity during its development and in adulthood. NCAM functions may be regulated by the addition of long linear homopolymers of alpha 2-8-linked sialic acid (PSA). PSA is attached to NCAM via either of two specific sialyltransferases: ST8SiaII and ST8SiaIV. PSA-NCAM is expressed abundantly in the retina and optic nerve during development and adulthood. In the retina PSA-NCAM is expressed in the glial cells in close proximity to retinal ganglion cell (RGC). The functions of the PSA-NCAM in the retina remain unknown. The aim of this study was to investigate the roles of PSA-NCAM in the survival of RGCs after administration of the exitotoxin kainic acid (KA). Intraocular administration of KA induced reduction in the density of RGCs approximately by 60%. Administration of endoneuraminidase (Endo-N) an enzyme, which removes PSA residues from the surface of NCAM, enhanced the toxic effect of KA on RGC. In knockout mice with constitutive deficiency of either ST8SiaII or ST8SiaIV genes, the levels of PSA-NCAM did not differ from those in wild type mice. The toxicity of KA on RGC in these animals also did not differ from control. It should be noted, however, that in knockout ST8SiaII-/- adult mice a reduced number of RGCs was found despite the presence of high levels of PSA-NCAM. These data suggest that during development ST8SiaII ensures high levels of PSA-NCAM, which necessary for the developmental survival of RGCs. The PSA-NCAM in the adult retina ensures the resistance of RGCs to injury.

